# Pre-clinical study of drug combinations that reduce breast cancer burden due to aberrant mTOR and metabolism promoted by LKB1 loss

**DOI:** 10.18632/oncotarget.2818

**Published:** 2014-11-25

**Authors:** Rafaela Andrade-Vieira, Donna Goguen, Heidi A. Bentley, Chris V. Bowen, Paola A. Marignani

**Affiliations:** ^1^ Department of Biochemistry and Molecular Biology, Dalhousie University, Faculty of Medicine, Halifax, Nova Scotia Canada; ^2^ Department of Radiology, Halifax, Nova Scotia Canada

**Keywords:** LKB1, HER2, pre-clinical, cancer, metabolism

## Abstract

Cancer therapies that simultaneously target activated mammalian target of rapamycin (mTOR) and cell metabolism are urgently needed. The goal of our study was to identify therapies that effectively inhibited both mTOR activity and cancer cell metabolism in primary tumors *in vivo*. Using our mouse model of spontaneous breast cancer promoted by loss of LKB1 expression in an ErbB2 activated model; referred to as LKB1^−/−^NIC mice, we evaluated the effect of novel therapies *in vivo* on primary tumors. Treatment of LKB1^−/−^NIC mice with AZD8055 and 2-DG mono-therapies significantly reduced mammary gland tumorigenesis by inhibiting mTOR pathways and glycolytic metabolism; however simultaneous inhibition of these pathways with AZD8055/2-DG combination was significantly more effective at reducing tumor volume and burden. At the molecular level, combination treatment inhibited mTORC1/mTORC2 activity, selectively inhibited mitochondria function and blocked MAPK pro-survival signaling responsible for the ERK-p90RSK feedback loop. Our findings suggest that loss of LKB1 expression be considered a marker for metabolic dysfunction given its role in regulating AMPK and mTOR function. Finally, the outcome of our pre-clinical study confirms therapies that simultaneously target mTORC1/mTORC2 and glycolytic metabolism in cancer produce the best therapeutic outcome for the treatment of patients harboring metabolically active HER2 positive breast cancers.

## INTRODUCTION

The metabolic branch of mammalian target of rapamycin (mTOR) signaling is primarily dependent on the energy sensing 5′ AMP-activated protein kinase (AMPK) and is under-utilized as a strategy to target aberrant mTOR signaling. The main activator of AMPK is the serine-threonine tumor suppressor kinase LKB1, which is responsible for phosphorylating AMPK on Thr172, leading to the activation of the energy sensor [1, 2]. Both somatic and sporadic mutations have been identified in LKB1 and are responsible for numerous malignancies [3]. Arguably, targeting activating mutations in the phosphoinoside-3-kinase (PI3K)/AKT pathway have proven to be a viable strategy for inhibiting mTOR, however, in cancers that are mutant for LKB1 [3], AMPK-mediated regulation of mTOR will be compromised. As such, the metabolic branch of mTOR signaling, mTORC1, will be hyperactive, particularly if there are associated activating mutations in oncogenes. Because hyperactive mTOR is often found in cancer associated with activating mutation in the PI3K/AKT signaling pathway, significant effort has been made to develop therapeutic strategies that target PI3K/AKT signaling for the treatment of cancer. Current treatment strategies are at various stages of clinical trial, specifically NVP-BEZ235, PF-04691502 and BKM120 [4-6]. These new compounds are promising however there may be limitations as these drugs are highly dependent on tumor sub-type, are specific to particular genetic alterations, and may lead to the activation of negative feedback loops that acerbate resistance or recurrence. By exclusively targeting activating mutations in the PI3K/AKT branch of mTOR signaling, it stands to reason that if these same cancers express mutation in LKB1 or express isoforms of the pseudokinase STRADα that render LKB1 catalytically deficient [7], the tumors may initially regress in response to treatment, however because AMPK activity is deregulated and the mTORC1-MAPK feedback loop is activated [8], the cancer will invariably return and/or be resistant to future treatments.

Recently we discovered that 31% of HER2 positive breast cancer lacked expression of LKB1 [9]. Based on this discovery we developed a mouse model of breast cancer where an activating mutation in the ErbB2 oncogene was combined with loss of LKB1 expression (LKB1^−/−^NIC mice) [9]. In this model, we observed that loss of LKB1 activity promoted tumor growth by significantly reducing the latency of ErbB2-mediated tumorigenesis. Furthermore, tumorigenesis was strongly associated with hyperactivation of mTOR and dysregulation of cell metabolism, giving rise to metabolically active tumors. We found that inhibition of mTOR with AZD8055, a novel ATP-competitive inhibitor of mTOR that inhibits both mTORC1 and mTORC2 [10], inhibited mTOR signaling and expression of glycolytic enzymes, lactate dehydrogenase (LDH) and pyruvate dehydrogenase (PDH), in primary breast cancer cells isolated from LKB1^−/−^NIC mice [9]. Further to this, we and others observed a reduction in LDH expression and therefore lactate, in response to inhibition of mTOR by Rapamycin [11, 12].

It is clear that alternative treatment strategies are necessary to overcome hyperactivated mTOR and dysregulation of cell metabolism attributed to the loss of LKB1 regulation of AMPK signaling pathways. Given that aerobic glycolysis plays a significant role in tumorigenesis, targeted regulation of ATP production may present as a viable option for the treatment of cancer. 2-deoxyglucose (2-DG) inhibits a rate-limiting step in glycolysis as it is taken up by the cell and metabolized by hexokinase to phospho-2-DG (p-2-DG), a competitive inhibitor of hexokinase [13, 14]. As a mono-therapy, 2-DG-mediated growth suppression appears to be offset by a concomitant induction of AKT activation through phosphorylation of Thr308 and Ser473 [15]. We conducted pre-clinical trials to investigate novel mono-therapies and combinatorial therapies that targeted mTOR and metabolism in mammary gland tumorigenesis. In our study, we specifically evaluated inhibition of both mTORC1 and mTORC2 with AZD8055 in combination with the glycolytic inhibitor 2-DG, in LKB1^−/−^NIC mice with aggressive primary breast cancer [9]. Using magnetic resonance imaging (MRI), we monitored changes in tumor growth in response to treatments and elucidated the role metabolism plays in our model. Herein we confirm that targeted combinatorial therapy that simultaneously inhibits mTOR signaling and glycolytic metabolism is a viable strategy for the treatment of aggressive primary breast cancer.

## RESULTS

### Loss of LKB1 expression enhances cell metabolism

**Figure 1:**
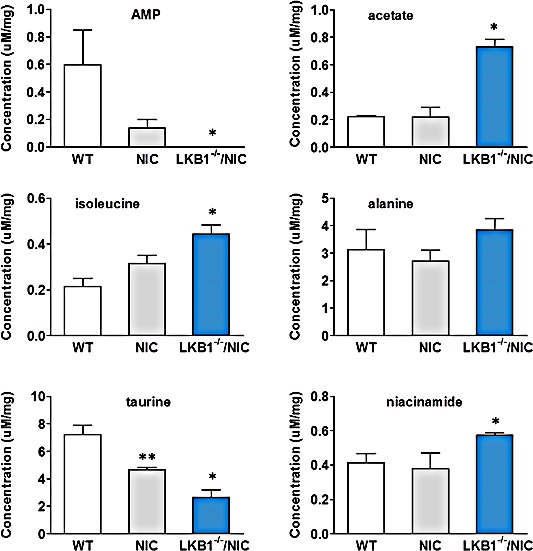
Enhanced metabolism in primary tumor cells lacking LKB1 expression NMR analysis of metabolites in LKB1^−/−^NIC mammary tumors, NIC tumors and wild-type (WT) mammary epithelial cells. For each group, data is reported as three separate samples (μM/mg) ±SEM, P<0.05. One-way ANOVA followed by Bonferroni post-hoc test for multiple comparisons and *P* values were calculated. *LKB1^−/−^NIC significantly different from NIC and WT, **NIC significantly different from WT.

We previously observed that ATP levels in LKB1^−/−^NIC primary mammary tumor cells were elevated compared to the level of ATP in wild-type mammary epithelial cells, as were other important metabolites quantified from whole tumors [9]. To determine whether loss of LKB1 expression is responsible for enhanced breast cancer cell metabolism, we analyzed the presence of metabolites in whole LKB1^−/−^NIC mammary tumors compared to tumors harvested from NIC control mice and from wild-type (WT) mammary glands by NMR analysis (Chenomx Inc.) (Fig.1). Normally, when cellular AMP levels are elevated, the binding of AMP to a AMPK enhances the substrate readiness of AMPK for phosphorylation/activation by the LKB1 complex, resulting in inhibition of mTOR signaling and fat synthesis [16, 17]. Thus, AMPK serves as a metabolic switch that senses the energy requirements of the cell. Metabolic analysis of AMP concentrations in LKB1^−/−^NIC tumors (0.001 ± 0.00 μM/mg), NIC tumors (0.13 ± 0.05 μM/mg) and WT (0.59 ± 0.14 μM/mg) mammary tissues indicate that AMP concentrations are significantly reduced in mammary tumors compared to WT tissue (P<0.05). Under these circumstances, AMPK activity in LKB1^−/−^NIC tumors would be compromised on two counts; loss of LKB1 activity and reduced AMP concentrations, whereas in NIC mice, AMPK activity would be less impacted since NIC tumors express LKB1 comparable to WT mammary tissues [9]. As a result, regulation of mTOR in LKB1^−/−^NIC mice is increased, as is the regulation of metabolic pathways. Of the biosynthetic metabolites that are necessary for the maintenance of cell metabolism and mitochondria function, acetate is used in cells as part of acetyl-CoA group and is the first molecule to enter the Krebs cycle. Acetate concentrations in LKB1^−/−^NIC tumors (0.73 ± 0.03 μM/mg), was significantly elevated compared to the concentration in NIC tumors or WT mammary tissue (0.22 ± 0.07 and 0.22 ± 0.01 μM/mg, respectively; P<0.05). The amino acids isoleucine and alanine are direct sources of ions for cell metabolism, as they are found in mitochondria and are precursors for the synthesis of key elements of the Krebs cycle and metabolic pathways. In LKB1^−/−^NIC tumors, isoleucine (0.44 ± 0.02 μM/mg) was significantly elevated compared to levels in NIC and WT gland (0.31 ± 0.03 and 0.21 ± 0.02 μM/mg, respectively; P<0.05). Alanine levels were modestly elevated in LKB1^−/−^NIC (3.84 ± 0.41 μM/mg) tumors compared to NIC (2.71 ± 0.23 μM/mg) and WT (3.13 ± 0.42 μM/mg), however the differences were not statistically significant. Taurine plays a role in antioxidant and anti-inflammatory pathways, with low levels of taurine implicated in a variety of metabolic diseases [18, 19]. In LKB1^−/−^NIC tumors, we observed a significant reduction in taurine (2.66 ± 0.31 μM/mg) concentration compared to NIC and WT gland (4.68 ± 0.13 and 7.23 ± 0.38 μM/mg, respectively; P<0.1). Niacinamide is incorporated into NAD coenzyme and is involved in a variety of mitochondria enzymatic reactions. In our model, niacinamide was significantly elevated in LKB1^−/−^NIC tumors (0.57 ± 0.007 μM/mg) compared to NIC and WT tissues (0.38 ± 0.05 μM/mg and 0.41 ± 0.03 μM/mg, respectively; P<0.05) (Fig. 1). These results suggest that the loss of LKB1 promotes ErbB2-mediated mammary tumorigenesis, in part, through metabolic processes since analysis of NIC tumors displayed less metabolic activity than tumors from LKB1^−/−^NIC.

### Inhibition of mTOR and PI3K impairs tumor growth

**Figure 2:**
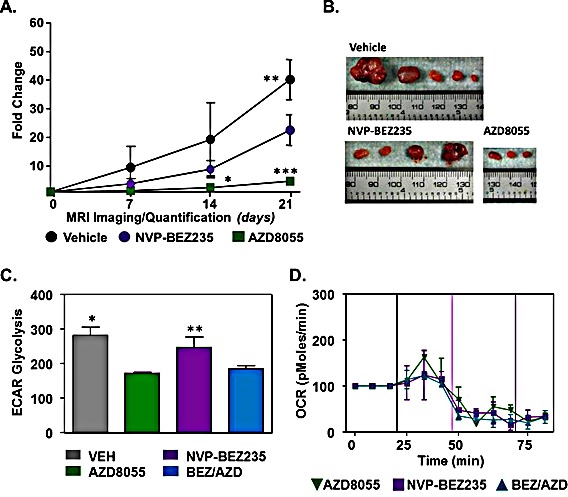
Effects of PI3K and mTOR inhibition on primary tumor development (A) 20 week old mice were treated with Vehicle, NVP-BEZ235 (10 mg/kg) and AZD8055 (20 mg/kg) daily for 21 days. Tumor volume was evaluated weekly by caliper measurements. Data represents mean of three independent mice ± SD, P<0.05. One-way ANOVA followed by Bonferroni post-hoc test for multiple comparisons and *P* values were calculated. *AZD8055 compared with Vehicle. **Vehicle compared with NVP-BEZ235, ***AZD8055 compared with NVP-BEZ235 and Vehicle. (B) Representative tumors excised from LKB1^−/−^NIC after 21 days of treatment with indicated drugs. (C) ECAR and (D) OCR measurements of primary mammary tumor cells isolated from LKB1^−/−^NIC treated with AZD8055, NVP-BEZ235 and BEZ/AZD. Data is representative of three separate mice per treatment group; mean ± SD, P<0.05, One-way ANOVA, followed by Bonferroni post-hoc test for multiple comparisons and *P* values were calculated. *Vehicle compared with AZD8055 and BEZ/AZD combination, **NVP-BEZ235 compared with AZD8055 and BEZ/AZD combination.

Having confirmed that the loss of LKB1 in our model is responsible for enhanced metabolic activity, we were interested in whether treatment of LKB1^−/−^NIC mice *in vivo* with compounds that target the PI3K pathway and mTOR would be effective at inhibiting tumor growth. LKB1^−/−^NIC mice at 20 weeks [9] received daily intraperitoneal (i.p.) administration for 21 days and tumor volume was determined weekly using caliper measurements. We observed that mice treated with NVP-BEZ235 (10mg kg^−1^) resulted in a significant reduction in tumor growth (22.58 ± 10.65, n=3 mean ± SD, P<0.01) by day 21 of treatment, compared with Vehicle treated mice (40.19 ± 6.97, n=3 mean ± SD) (Fig. 2A, B). We treated mice with the mTOR inhibitor AZD8055 (20mg kg^−1^) and found that inhibition of mTORC1 and mTORC2 significantly inhibited tumor growth (4.72 ± 1.19, n=3 mean ± SD, P<0.001) compared with Vehicle treated mice (Fig. 2A, B). Further to this, tumor volume in response to AZD8055 treatment was significantly reduced compared with tumor volume in response to NVP-BEZ235 treatment (P<0.01) (Fig. 2A, B). Tumor volume in response to treatments was similar up to day 14, after which there was a significant impairment in tumor growth in response to AZD8055 treatment compared with Vehicle treatment (2.5 ±0.9 and 19.29 ±12.8, n=3 mean ± SD, P<0.01 respectively) (Fig. 2A).

### The effects of drug therapy on mitochondria function

Previously, we showed that treatment of primary breast cancer cells isolated from LKB1^−/−^NIC mice with AZD8055 significantly inhibited mTORC1/mTORC2, as well as inhibition of glycolytic enzymes identified as drivers of the Warburg effect [9]. To determine whether mitochondria function is altered in our model, we treated LKB1^−/−^NIC primary breast cancer cells using AZD8055 (100 nM) alone, NVP-BEZ235 (100 nM) alone and combination AZD8055/NVP-BEZ235 (100 nM/100 nM) followed by analysis of aerobic glycolysis (Fig. 2C) and oxygen consumption rates (Fig. 2D). Using the Seahorse XF24 analyzer, we observed that extracellular acidification rate (ECAR), a marker of aerobic glycolysis, was significantly decreased in response to both AZD8055 treatment alone (172 ± 5.2 mpH/min) and NVP-BEZ235 + AZD8055 combination treatment (184.3 ± 14.8 mpH/min) compared with NVP-BEZ235 treatment alone (246.7 ± 51.2 mpH/min; **P<0.05) and Vehicle (281.3 ± 24.0 mpH/min; *P<0.05). Aerobic glycolysis in NVP-BEZ235-treated cells was not different from aerobic glycolysis in Vehicle- treated cells (Fig. 2C). In the same experiments, oxygen consumption levels were found to be decreased in response to mono- and combination therapies, indicative of decreased metabolic function (Fig. 2D). Collectively, this data suggests that both AZD8055 and NVP-BEZ235 mono-therapy decreased tumor growth in LKB1^−/−^NIC mice, however the inhibition of mTOR by AZD8055 was significantly more effective at preventing tumor growth compared with NVP-BEZ235 treatment alone. Given that NVP-BEZ235 is a poor inhibitor of AKT and PDK1 [20, 21], and inhibition of mTOR by AZD8055 prevents the activation of both AKT-T308 and AKT-S473 [9], in our model AZD8055 is a better treatment for breast cancer.

### Inhibition of tumor growth, in response to 2-DG and AZD8055 treatments

**Figure 3:**
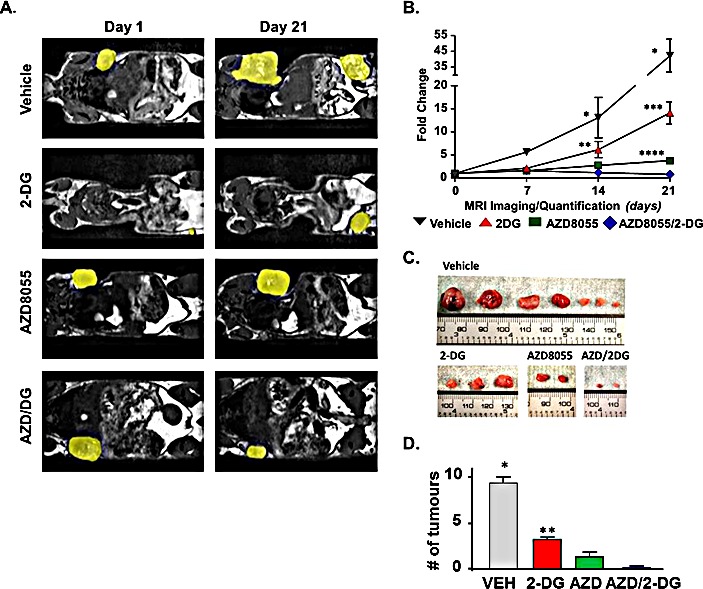
Pre-clinical evaluation of AZD8055 plus 2-DG combination treatment (A) At 20 weeks of age, mice were treated with Vehicle, AZD8055 (20 mgkg^−1^) alone, 2DG (25 mgkg^−1^) alone or a combination of AZD8055 plus 2DG daily (i.p.) for 21 days. Primary tumors were visualized by MRI every 7 days and tumor volume was quantified. Yellow patches highlight mammary tumors. (B) Changes in tumor volume are represented as fold change from Day 0, start of treatment. Data is representative of five - eight mice per treatment group ± SD, *P<0.0001 Vehicle compared to all treatments, **P<0.01 2-DG compared to Vehicle. ***P<0.0001 2-DG compared to AZD8055 and combination. ****P<0.01 AZD8055 compared to combination. Two-way ANOVA followed by Bonferroni post-hoc test for multiple comparisons and *P* values were calculated. (C) Representative tumors excised from LKB1^−/−^NIC mice after 21 days of treatment with indicated drugs. (D) The average number of mammary tumors per LKB1^−/−^NIC mouse after 21 days of treatment. Data is representative of three separate mice per treatment group ± SD, **P<0.01. One-way ANOVA followed by Bonferroni post-hoc test for multiple comparisons and *P* values were calculated.

Having shown that treatment of LKB1^−/−^NIC primary mammary tumor cells with AZD8055 inhibited key glycolytic enzymes, namely PDH and LDH, we wanted to explore beyond our previous *ex vivo* findings [9]. Because mTOR is a regulator of aerobic glycolysis by promoting activation of glycolytic enzymes [22], we evaluated whether it was feasible to simultaneously inhibit glycolysis and mTOR activity in LKB1^−/−^NIC mammary tumors by treating mice daily for 21 days with low dose 2-DG (25 mgkg^−1^) alone, AZD8055 (20 mgkg^−1^) alone and 2-DG plus AZD8055 (25mgkg^−1^plus 20 mgkg^−1^). For these longitudinal studies, mice were pre-screened by magnetic resonance imaging (MRI) at 19 weeks of age to identify early tumor bearing mice, after which treatments were initiated at week 20 with daily injections (i.p) for 21 days. Treatment duration was determined by ethical endpoint tumor burden of 10% body weight. Treatments were well tolerated by the mice for the duration of the study and no variation in weight gain was observed. Tumor volume was measured by MRI every seven days with representative mice shown at Day 0 and Day 21 for each treatment (Fig. 3A). We did not observe any differences in tumor volume after treatment for seven days (Fig. 3B); however by 14 days of treatment, tumor volumes were significantly reduced in response to AZD8055 alone and in combination treatment, compared with treatment with 2-DG alone or Vehicle. Compared with Day 0, Vehicle treatment for 21 days resulted in an increase in tumor volume by 42.3 ±10.4 fold, whereas both AZD8055 alone and 2-DG alone significantly inhibited tumor growth by Day 21, compared with Day 0 (3.7 ± 1.6 and 14.1 ± 2.4 fold, respectively) (Fig 3A-B). Compared with start of treatment, the combination of AZD8055 + 2-DG significantly decreased tumor volume (0.85 ± 0.4 fold) by Day 21 compared with Vehicle (42.3 ±10.4 fold), 2-DG treatments (14.1 ± 2.4 fold) and AZD8055 (3.7 ± 1.6 fold) mono-therapies (Fig 3. A-B). Tumors were harvested at the end of 21 days and in agreement with MRI volumetric analysis, tumors were consistently smaller from combination treated mice compared to mono-therapies and Vehicle treatments (Fig. 3C). Furthermore, tumor burden in response to treatment was significantly different between 2-DG treated mice and those treated with AZD8055 alone or combination treatment (Fig. 3D).

### Mitochondria content shifts in response to AZD8055 and 2-DG

**Figure 4:**
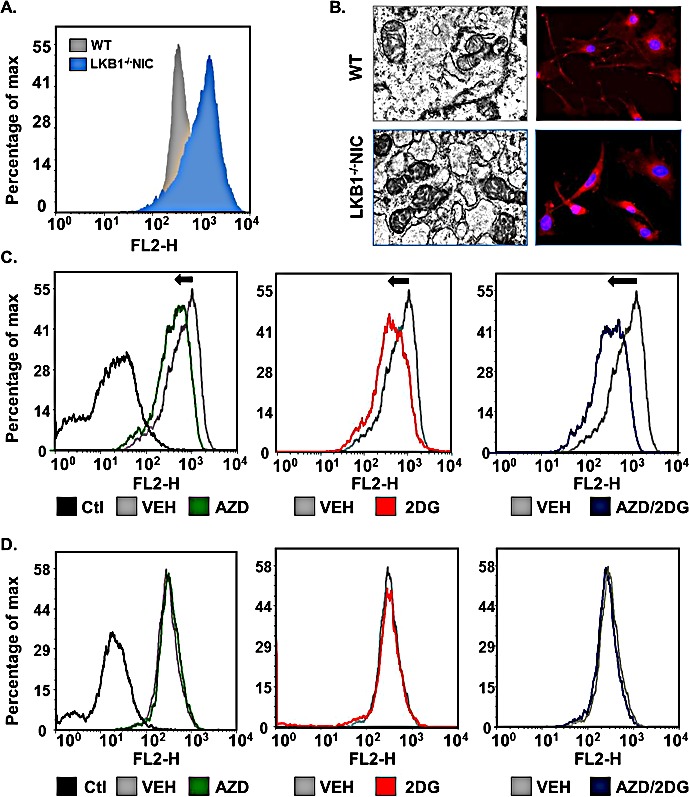
Primary mammary tumor cells undergo metabolic shifts in response to treatments Mitochondria content was assessed using mitotracker Red. (A) Primary mammary epithelial tumor cells were isolated from LKB1^−/−^NIC mice and compared to mammary epithelial cells isolated from wild-type mice. (B) Transmission electron microscopy showing mitochondria (left image) and Fluorescence Red CMX-ROS (right image) were used to visualize mitochondria. DAPI was used to visualize nuclei. (C) Mammary tumor epithelial cells isolated from LKB1^−/−^NIC mice and (D) Mammary epithelial cells isolated from wild-type mice were treated for 2h with indicated drugs and incubated with mitotracker for 20 min. Representative histograms of mitochondria content are shown for LKB1^−/−^NIC (C) and WT (D). Non- stained cells are displayed as negative control (Ctl). Data is representative of three separate mice for each treatment group.

To determine whether there was a difference in mitochondria function between LKB1^−/−^NIC primary mammary tumor cells and WT mammary epithelial cells, we measured mitochondria content in LKB1^−/−^NIC and WT cells using mitotracker red CMX/ROS (Fig. 4A). We observed that LKB1^−/−^NIC primary mammary tumor cells had greater mitochondria content than WT mammary epithelial cells. In addition, we analyzed mitochondria morphology by transmission electron microscopy and fluorescent microscopy (Fig. 4B) and found that morphologically, mitochondria in LKB1^−/−^NIC cells were enlarged with increased cristae density compared to mitochondria in WT mammary epithelial cells, suggestive of increased ATP-production capacity.

To determine the effect of inhibiting mTOR and/or glycolysis on mitochondria content, we treated cells with AZD8055, 2-DG and AZD8055 + 2-DG for 2 hours, followed by analysis of mitochondria metabolic activity. In LKB1^−/−^NIC cells, both mono- and combination therapies resulted in a shift in mitochondria content to the left; indicative of reduced mitochondria content, compared with Vehicle-treated LKB1^−/−^NIC cells (Fig. 4C). In response to combination treatment, we consistently observed a greater reduction in mitochondria content compared with mono-therapies. For WT mammary epithelial cells, we did not observe any difference in mitochondria content between mono-therapies, combination therapy, or Vehicle treatment (Fig. 4D). These results suggest that both AZD8055 and 2-DG as mono-therapies or in combination are well tolerated by mitochondria, as not altering mitochondria biogenesis of normal cells. Furthermore, these results suggest that loss of LKB1 signaling in breast cancer reduces the ability of cells to overcome metabolic stress; however, treatments that target aberrant glycolysis and mTOR signaling drive the cells to overcome metabolic stress, ultimately resulting in decreased tumourigenesis, as observed in our pre-clinical study (Fig. 3).

**Figure 5:**
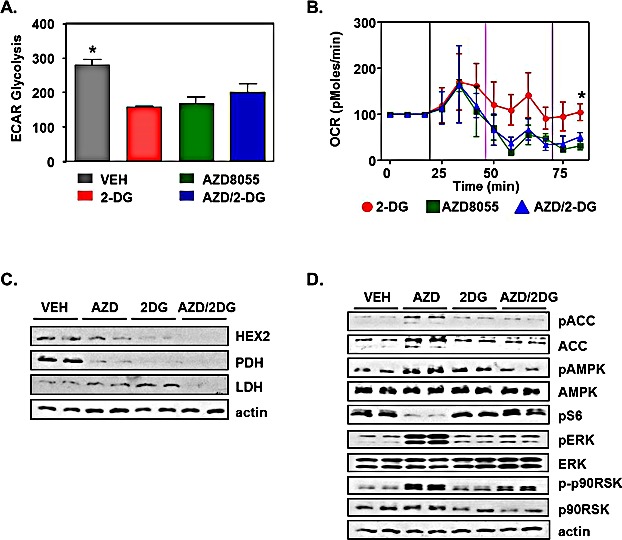
Pro-survival pathways inhibited in response to treatment Primary mammary tumor cells were isolated from LKB1^−/−^NIC mice, treated with Vehicle (VEH), AZD8055, 2-DG and AZD/2-DG combination, followed by analysis of mitochondria function. (A) Acidification rates (ECAR) was determined using cells isolated from three separate mice per treatment group, in duplicate; mean ± SEM, P<0.05, One-way ANOVA, followed by Bonferroni post-hoc test for multiple comparisons and *P* values were calculated. *VEH compared with AZD8055, 2-DG and AZD/2-DG combination. (B) Oxygen consumption rate (OCR) was determined using cells isolated from three separate mice per treatment group, in duplicate; mean ± SEM, P<0.05, One-way ANOVA, followed by Bonferroni post-hoc test for multiple comparisons and *P* values were calculated. *2-DG treatment compared with AZD8055, and AZD/2-DG combination. (C) LKB1^−/−^NIC mice were treated for 21 days with VEH, AZD8055, 2DG, and combination of AZD/2-DG. Mammary tumors were harvested and proteins were prepared for western blot analysis. Duplicate protein samples were loaded and analyzed. Data is representative of three separate mice per treatment. (D) LKB1^−/−^NIC mice were treated for 21 days with VEH, AZD8055, 2DG, and combination AZD/2-DG. Tumors were harvested and proteins were prepared for western blot analysis. Duplicate samples were loaded and analyzed. Data is representative of three separate mice per treatment.

Given that we observed a shift in mitochondria content in response to AZD8055 alone, 2-DG alone and combinatorial therapy, we investigated whether there were corresponding changes in mitochondria function that would challenge the energy requirements for rapidly growing tumors. We investigated the capacity of LKB1^−/−^NIC primary mammary tumor cells for aerobic glycolysis (ECAR), and oxygen consumption rate (OCR) in response to treatments. Using the Seahorse XF analyzer, we treated LKB1^−/−^NIC primary mammary tumor cells using 2-DG, AZD8055 and combination of both. We observed that treatment of cells with 2-DG alone (160.0 ± 1.5 mpH/min), AZD8055 alone (169 ± 17.9 mpH/min) and combination treatment (200.7 ± 26 mpH/min) significantly inhibited ECAR levels compared to Vehicle treatment (281.3 ± 13.8 mpH/min; P<0.05) (Fig. 5A). Treatment of cells with AZD8055 alone or combinatorial treatment significantly reduced OCR compared with 2-DG treatment alone (Fig. 5B). This data strongly supports that combination treatment of AZD8055/2-DG reduces mitochondria function and aerobic glycolysis in LKB1^−/−^NIC mammary tumor cells.

As tumors develop, faulty metabolic switches allow for growth advantages over normal cells. mTOR enhances transcription of glycolytic enzymes, thereby increasing the capacity of glycolysis metabolism [23-25]. Thus, the inhibition of mTOR would be a strategy for controlling glycolytic metabolic switches. Further to this, the inhibition of the rate-limiting enzyme hexokinase 2 (HEX2) could augment the effects of mTOR inhibition. To explore whether mono- or combination therapies altered the expression of glycolytic enzymes in LKB1^−/−^NIC mammary tumors, we analyzed expression of HEX2, LDH and PDH in whole tumors harvested from mice that had been treated with AZD8055, 2-DG, combination treatment and Vehicle for 21 days (Fig. 5C), by western blot analysis. AZD8055 treatment of LKB1^−/−^NIC mice modestly reduced expression of HEX2 compared with 2-DG treatment *in vivo* however; combination therapy exhibited the strongest inhibitory effect on HEX2 expression. LDH expression was reduced only in response to combination therapy, compared with mono-therapies, while all treatments inhibited expression of PDH compared with Vehicle treatment (Fig. 5C). These results suggest that treatment of LKB1^−/−^NIC mice with AZD8055 in combination with 2-DG, leads to molecular changes in metabolic switches that contribute to inhibition of tumourigenesis (Fig. 3).

Previous work by others has shown that prolonged inhibition of either PI3K or mTOR by drugs or by genetic means, leads to activation of a negative feedback loop that activate MAPK signaling [8, 26, 27]. As such, we examined the effect of mono-therapies and combination therapy on the expression of both AMPK and MAPK signaling pathways in whole mammary tumors from LKB1^−/−^NIC mice by western blot analysis (Fig. 5D). In response to AZD8055 treatment for 21 days, tumor volume and burden were significantly reduced (Fig. 3), while the expression of pACC, the direct target of AMPK, and pAMPK were elevated, and expression of pS6, the target of S6 kinase, was markedly reduced, compared with other treatments. As expected [8, 26, 27], the phosphorylation status of p90RSK and ERK were elevated in response to prolonged inhibition of mTOR by AZD8055, compared with other treatments (Fig. 5D). Given that 2-DG is a known activator of pro-survival pathways via PI3K and insulin-like growth factor receptor 1 [14, 28], in our model, both tumor volume and burden were significantly reduced, compared with Vehicle treatment (Fig. 3), however the phosphorylation status of S6, ACC and AMPK were unchanged from Vehicle-treated mice. The phosphorylation status of p90RSK and ERK was also unchanged compared to Vehicle-treated (Fig. 5D). Finally, treatment of LKB1^−/−^NIC mice for 21 days with combination therapy showed the greatest impact on tumor volume and burden (Fig 3) with no change in the phosphorylation status of S6 compared with 2-DG treatment alone and Vehicle-treated mice (Fig. 5D). Interestingly, the phosphorylation status of ERK and RSK were unchanged in Vehicle-treated mice despite the increase observed in response to AZD8055 mono-therapy. These results suggest that the simultaneous inhibition of mTORC1/mTORC2 and glycolytic pathways prevents the mTORC1 negative feedback loop and likely enhances both AZD8055- and 2-DG-mediated growth inhibition since activation of the MAPK pathway is prevented.

## DISCUSSION

In the present study we evaluated the effect of mono-therapies and combination therapy on spontaneous primary mammary tumors from LKB1^−/−^NIC mice that we previously characterized as hyperactive for mTOR and enhanced metabolic activities promoted by the loss of LKB1 expression and gain of ErbB2 function [9]. Our LKB1^−/−^NIC mouse model is representative of spontaneous primary human breast cancers that are HER2 positive with deregulated metabolic activity. Initial characterization *ex vivo* of primary mammary tumor cells confirmed that inhibition of mTOR by AZD8055 significantly reduced AKT, mTORC1/mTORC2 and glycolytic activities [9]. Pre-clinical longitudinal studies that targeted the PI3K and p70S6K pathways with competitive NVP-BEZ235 inhibitor was not as effective at reducing tumor volume and burden as targeting mTOR with AZD8055 or glycolysis with 2-DG mono-therapies. A plausible explanation for this difference may be attributed to the fact that NVP-BEZ235 is a poor inhibitor of AKT and PDK1 [20, 21], whereas AZD8055 inhibition of mTOR prevents the activation of both AKT-T308 and AKT-S473 [9]. Thus in our model, AZD8055 is a better treatment.

Recently, LKB1 inactivation has been shown to cooperate with activating oncogene mutations to drive tumor progression in various models of cancer [9, 26, 29, 30]. The primary substrate of LKB1 is the central regulator of energy homeostasis and metabolic checkpoint, AMPK. At the nexus between growth factor receptor signaling and cellular energy metabolism, AMPK when activated regulates protein and lipid synthesis, inhibits mTORC1 through activation of tuberous sclerosis complex 2 (TSC2) and phosphorylation of raptor [1, 31-33]. When LKB1-AMPK signaling is functional, regulation of the metabolic branch of mTOR signaling is intact regardless of whether PI3K/AKT or receptor tyrosine kinase signaling is aberrant. In our LKB1^−/−^NIC mouse model of primary breast cancer, LKB1-AMPK signaling is significantly compromised, thus mono-therapy with the dual ATP-competitive PI3K/mTOR inhibitor NVP-BEZ235 was insufficient to block tumorigenesis whereas the combination of NVP-BEZ235 and AZD8055 resulted in reduced mitochondria function comparable to AZD8055 mono-therapy alone. Unlike NVP-BEZ235, AZD8055 inhibits phosphorylation of mTORC1 and mTORC2 substrates, namely p70S6K and 4E-BP1, leading to significant inhibition of cap-dependent translation, and phosphorylation of AKT at residues S473/T308, respectively [9, 10]. Our pre-clinical data strongly suggest that treatment with AZD8055 as a mono-therapy is sufficient to inhibit primary mammary gland tumorigenesis in LKB1^−/−^NIC mice, however the negative feedback loop that leads to the activation of MAPK signaling [8, 26, 27] could lead to relapse. Thus, a novel combinatorial approach that targets metabolic processes is warranted.

Therapies that activate the AMPK signaling pathway such as the biguanides metformin and phenformin have been used for the treatment of diabetes. Given the role these compounds play in regulated glycolytic metabolism as well as mitochondria function [34, 35], clinical trials are underway for the treatment of cancer, highlighting the importance of targeting cancer cell metabolism in metabolically active diseases. Since aerobic glycolysis is a major source of energy and provides biosynthetic products for protein and lipid synthesis, targeted inhibition of glycolysis would ultimately impact tumor growth. Inhibition of the rate-limiting step in glycolysis with 2-DG leads to depletion of ATP [13, 14], potentially shifting the Warburg Effect [36]. Results from Phase II clinical trials using 2-DG for the treatment of osteosarcomas and lung cancer, suggest that mono-therapy with 2-DG may not be as promising as combinatorial therapy, however in combination with other targeted treatments such as paclitaxel, inhibition of glycolysis with 2-DG sensitized tumors to the chemotherapeutic agents [28]. In another study, treatment of LNCaP prostate cancer cells with 2-DG in combination with the AMPK activator metformin, resulted in activation of pro-death pathways *in vitro* inducing p53-dependent apoptosis via the energy sensor pathway AMPK [37].

**Figure 6:**
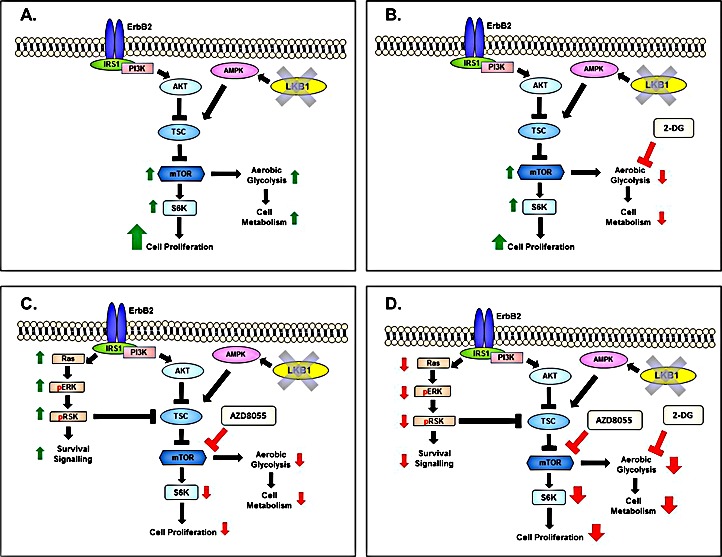
Inhibition of mTOR and Aerobic Glycolysis in LKB1NIC Mouse Model of Breast Cancer (A) Schematic representation of signaling pathways previously characterized in LKB1^−/−^NIC mice [9]. Loss of LKB1 expression in ErbB2/HER2 mouse model of primary breast cancer leads to hyperactivation of mTOR and enhanced cancer cell metabolism. (B) Cell signaling in LKB1^−/−^NIC mammary gland tumors in response to 2-DG treatment. Schematic shows inhibition of aerobic glycolysis leading to decreased cell metabolism. 2-DG treatment does not affect mTOR activity, therefore in absence of LKB1 expression and presence of ErbB2/HER2, mTOR remains hyperactive. (C) Cell signaling in LKB1^−/−^NIC mammary gland tumors in response to AZD8055 treatment. Schematic shows inhibition of mTOR signaling thereby preventing phosphorylation of S6K, inhibition of cell proliferation and a decrease in aerobic glycolysis due to low expression of glycolytic enzymes. Prolonged inhibition of mTOR leads to activation of pERK-p90RSK, activating pro-survival pathways. (D) Cell signaling in LKB1^−/−^NIC mammary gland tumors in response to combination treatment with AZD8055/2-DG. Schematic shows simultaneous inhibition of mTOR and aerobic glycolysis. In this model, glucose enters the cell but is not completely metabolized in response to 2-DG treatment. As such, pro- survival pathways that would be activated in response to prolonged inhibition of mTOR by AZD8055 treatment are suppressed via IRS1 signaling. The simultaneous inhibition of aberrant mTOR signaling and cell metabolism by AZD8055 and 2-DG leads to a significant reduction in tumor growth, burden and aberrant signaling pathways.

In our study, treatment of LKB1^−/−^NIC mice that are hyperactive for mTOR and metabolically active (Fig. 6A) with 2-DG mono-therapy (Fig. 3 and 6B) inhibited tumorigenesis, such that both tumor volume and burden were reduced in response to reduced aerobic glycolysis and mitochondria function, however AZD8055 mono-therapy (Fig. 3 and 6C) was significantly better at inhibiting tumor volume, burden and mitochondrial function. A possible explanation as to why 2-DG underperforms may be due to concomitant induction of AKT thereby activating pro-survival pathways [14, 38, 39]. With this in mind, prolonged treatment of LKB1^−/−^NIC mice with AZD8055 alone or 2-DG alone have challenges, in that both mono-therapies lead to enhanced activity of pro-survival pathways. However combining both AZD8055 and 2-DG treatment for 21 days synergized treatment effects, overriding pro-survival pathways, with the greatest effect on tumor volume, burden and mitochondria function. Thus, targeted treatment of hyperactive mTOR and aberrant glycolysis with combination therapy, AZD8055/2-DG inhibited the activation of ERK-mediated survival pathways associated with prolonged inhibition of mTOR [8, 40], as well as activation of AKT associated with 2-DG treatment [14, 38, 39] (Fig.3 and 6D). Interestingly, we observed phosphorylation of S6 in response to 2-DG monotherapy and combination therapy (Fig. 5C), despite inhibition of pro- survival pathways. A possible explanation for this could be that loss of LKB1 expression and therefore catalytic function, may contribute to the phosphorylation of S6 under an energy depletion state. Our findings are in agreement with work by others that evaluated 2-DG treatment of non-small cell lung cancer cells (NSCLC) that lacked expression of LKB1 compared to NSCLC cells that express LKB1 [41]. In this study, treatment of cells that lacked the expression of LKB1, with 2-DG, did not reduce the phosphorylation status of S6 compared to the phosphorylation status of S6 in cells that express LKB1 [41]. It is also known that phosphorylation of TSC2 by AMPK is required for translation regulation and S6 phosphorylation events [42] and more recently, it has been shown that activated AMPK is necessary for 2-DG mediated inhibition of pS6 [43]. Therefore the loss of LKB1 expression leads to loss of AMPK activation and impaired AMPK-mediated activation of TSC2 in response to energy deprivation caused by 2-DG treatment, emphasizing the importance of LKB1 in the regulation of energy homeostasis.

Mitochondria content was significantly reduced in response to combination treatment of LKB1^−/−^NIC primary mammary tumor cells, compared with mitochondria content of wild-type primary mammary epithelial cells that express a functional LKB1-AMPK signaling pathway (Fig. 4C). Since LKB1 and AMPK are necessary for biogenesis, cells lacking the LKB1-AMPK pathway would be more sensitive to treatment with these compounds as mono-therapies and/or in combination, while mitochondria content remains intact in WT mammary epithelial cells. These results suggest that AZD8055 and/or 2-DG compounds would not be detrimental to mitochondria function in normal cells when administered systemically.

In conclusion, we conducted pre-clinical trials using our LKB1^−/−^NIC mice to test mono-therapies and combinatorial therapies that targeted mTORC1/mTORC2 and metabolism in primary tumors. We discovered that treatment of mice with AZD8055 or 2-DG as mono-therapies was more effective at inhibiting mammary gland tumorigenesis than targeting PI3K pathways with competitive NVP-BEZ235 inhibitor. We also demonstrate that metabolically active cancer cells are more susceptible to the effects of metabolic drugs then normal mammary epithelial cells. We discovered that simultaneous inhibition of mTOR and metabolism with AZD8055 plus 2-DG combination was significantly more effective at inhibiting tumorigenesis and preventing sustained tumor growth that would occur through the activation of MAPK survival pathways. The outcome of our pre-clinical study emphasizes that mono-therapies directed towards ErbB2/HER2, PI3K or hyperactive mTOR alone, may not be adequate and sufficient to cause complete regression of primary tumors and/or prevent resistant phenotypes from developing. Our discovery strongly supports the practice of evaluation of LKB1 expression in tumors as a marker for aberrant mTOR and metabolic signaling. Furthermore, our model of primary breast cancer can be used as a tool to study the effectiveness of novel mono-therapies and combination therapies that are directed towards cancer that are hyperactive for mTOR and aberrant cancer metabolism. Future studies could take into consideration the role hyperactive mTOR and metabolism play in metastatic disease, and how best to treat this more lethal form of breast cancer and whether cell autonomous mechanisms are involved. As such, combination therapies that simultaneously target these pathways will provide the best clinical outcome for the treatment of metabolically active breast cancer.

## MATERIAL AND METHODS

### Animals

All animal husbandry and studies were conducted in strict accordance with the Canadian Council on Animal Care. Protocols #12-091, #13-063 were approved by the University Committee on Laboratory Animals (UCLA), Dalhousie University. LKB1*^fl/fl^*, NIC and LKB1*^−/−^*/NIC were as previously described [9]. Female mice were palpated every three days after week 18 to monitor for mammary tumors and weight change.

### Primary tumors and cells

Primary mammary tumors, primary mammary tumor cells and primary mammary epithelial cells were harvested from LKB1*^−/−^*/NIC and wild-type control mice as previously described [9]. Primary mammary epithelial cells were harvested and immediately used for experimentation.

### Magnetic Resonance Imaging

MRI images were acquired using a 3T Agilent MRI system specifically optimized for pre-clinical imaging, using a 21cm Magnex gradient coil and 25mm quadrature RF coil from Doty Scientific. MRI acquisitions involved 150 micron isotropic resolution 3D balanced-SSFP optimized for fat-tumor contrast. Volumetric tumor estimates were obtained through ellipsoidal model estimates in 3-plane orthogonal views using Agilent's VNMRJ package to measure tumor extent in each of 3 orthogonal directions. 3D rendered tumor volume calculations were performed using the RView software.

### Kinase Inhibitors

AZD8055 and NVP-BEZ235 were from Selleck, Chemicals. AZD8055 and NVP-BEV235 treatment protocols were based on previous drug studies conducted in the LKB1 conditional mice [26, 32, 44, 45]. 2-DG was from Toronto Research Chemicals. 2-DG treatments were based on studies conducted in various conditional mouse models [28, 46, 47].

### Western blot analysis

Following drug treatments, mammary tissues from wild-type animals and tumors from LKB1^−/−^NIC mice were harvested and protein lysates were prepared as previously described [9, 48]. The following antibodies were used: phospho-ribosomal protein S6 (pS6)(S235/236), -ACC, -pACC (S79), -pAMPK (T172), –AMPK, -pERK (T202/Y204), -ERK and antibodies against glycolytic proteins (all from Cell Signaling), -phospho-p90RSK (S380) and –p90RSK (Abcam), anti-LKB1 (Ley 37D), actin (Santa Cruz Biotechnologies, Santa Cruz, CA). Proteins were visualized by chemiluminescence (ECL) as previously described [49].

### Metabolic Assays

Extracellular acidification rate (ECAR) and oxygen consumption rate (OCR) were measured using XF24 analyzer (Seahorse Bioscience) as previously described [50]. Primary LKB1^−/−^NIC mammary tumor cells were isolated, then plated at 1×10^4^ per well in Dulbecco modified Eagle's medium (DMEM).

### Mitochondria Content

Primary LKB1^−/−^NIC mammary tumor cells were isolated as previously described [50] and plated onto coverslips. Treatments were performed using AZD8055 (100 nM), 2-deoxyglycose (10 mM) and a combination of both. Following this, cells were incubated for 20 min at 37°C with Mitotracker Red CMX/Ros (Cell Signaling) at a final concentration 100 nM. Cells were then washed 2x with PBS and prepared for analysis by fluorescence microscopy and flow cytometry. Fluorescence microscopy was conducted using a Nikon Eclipse TE 2000-E, mounted with a Q-Imaging CCD camera. Fluorescent images were acquired using Simple PCI software as previously described [3, 49]. Flow cytometry was conducted using a Becton Dickinson FACS Calibur. Data was acquired using CellQuest software and analyzed using ModFitLT as previously described [49].

### Electron microscopy

Cells were maintained as described above and collected for electron microscopy. Samples were fixed as previously described [51]. Once embedded in 100% Epon Araldite Resin, the samples were placed in a 60°C oven for 48 hours to cure thoroughly. Thin sections were cut using a LKB Huxley Ultramicrotome with a diamond knife and placed on 300 mesh copper grids. The grids containing the samples were stained, first with 2% × Aqueous Uranyl Acetate for 10 minutes, followed with 2 × 5 minute distilled water rinses. Lead citrate was then added for 4 minutes, followed with a quick rinse with distilled water. Samples were then allowed to air dry and viewed using a JEOL JEM 1230 Transmission Electron Microscope at 80kV. Images were captured using a Hamamatsu ORCA-HR digital camera.

### Statistics

Experiments were conducted in a minimum of three and reported as mean ± SD. The statistical analysis was performed by repeated measures one-way ANOVA, followed by Newman-Keuls or Bonferroni multiple comparison test. Values were statistically significant at p < 0.05, < 0.01, or < 0.0001 as indicated. The statistical analysis was performed using GraphPad Prism software 5.
